# Intervention to Prevent Recurrent Intestinal Parasitic Infections in People Living with HIV in Selected Parts of Eastern Cape, South Africa

**DOI:** 10.3390/tropicalmed9120289

**Published:** 2024-11-27

**Authors:** Ifeoma Anozie, Mojisola Clara Hosu, Teke Apalata, Dominic T. Abaver

**Affiliations:** 1 Department of Public Health, Walter Sisulu University, Mthatha 5117, South Africa; ifyanozie2002@yahoo.com; 2 Department of Laboratory Medicine and Pathology, Walter Sisulu University, Mthatha 5100, South Africa; tapalata@wsu.ac.za (T.A.); dabaver@wsu.ac.za (D.T.A.); 3 National Health Laboratory Service, Mthatha 5099, South Africa; 4 HERENDA Program, Department of Laboratory Medicine and Pathology, Walter Sisulu University, Mthatha 5100, South Africa

**Keywords:** HIV, hygiene education, intervention, intestinal parasitic infections, PLHIV, recurrent infections, sanitary practices

## Abstract

Interactions between parasites and hosts are not fully understood, though the dynamic pattern of infection and reinfection in humans varies with different demographic variables and behavioral changes. A community-based non-equivalent control group post-test-only design, an aspect of quasi-experimental design (QED), was carried out between March 2019 and February 2020. For the extraction of data from respondents, structural questionnaires were filled. Their CD4 count and viral load from the database of the National Health Laboratory Services, Mthatha were recorded. The method applied for the identification of intestinal parasites was a direct examination of the stool and the use of concentration methods. The post-test analysis showed that the intervention sites that received THEdS (Treatment, Health education, and Sanitation) bundle had a cure proportion of 60% and a re-infection proportion of 40%. The post-test results on control sites (treatment-only group) showed that the cure proportion was 51.4% and the re-infection proportion was 48.6%. The viral load significantly reduced from 377 to 44 copies/mL with a significant increment in CD4 count from 244 to 573 (cells µL) and (*p*-value) = 0.002. The combination of THEdS is an effective measure to reduce infection and reinfection of intestinal parasites. The THEdS bundle is a sustainable control and prevention method for the control of helminthes and protozoan associated with unsanitary environment and poor personal hygiene among immune-compromised individuals like HIV/AIDS patients.

## 1. Introduction

HIV/AIDS is a worldwide health emergency that has plagued the current century, and as of yet, no cure has been developed [1]. In 2023, the UNAIDS Global HIV and AIDS Statistics reported that an estimated 39.9 million individuals worldwide were living with HIV [2].

With an estimated 67% of the 38.4 million people living with HIV (PLHIV) worldwide in 2021, Sub-Saharan Africa (SSA) is the epicenter of the HIV/AIDS pandemic. SSA is also responsible for 280,000 of the 650,000 AIDS-related fatalities and 670,000 of the 1.5 million new infections that were recorded worldwide [3,4,5,6].

In 2022, about 12.4% of the entire population of South Africa, which is about 7.5 million, had HIV/AIDS [2,7]. A considerable segment of society is living in areas noted for impoverishment, rurality, and underdevelopment, and these are circumstances that have been associated with the transmission of HIV and parasitic illnesses. There is evidence that a significant relationship exists between intestinal parasitic infection, HIV, and other infections associated with immune-compromised individuals. Not only do these intestinal parasites cause chronic immune activation, but they also swing the immune response towards T-helper 2 responses [8]. Intestinal parasitic infections (IPI) in HIV-infected patients, which are within the spectrum of opportunistic infections, exacerbate their conditions causing morbidity and mortality and results in low CD4 count and compromised immune system [9].

IPI, caused primarily by these unicellular protozoans or multicellular helminths represent prevalent parasitic diseases which account for substantial morbidity in poorly resourced environments or countries with a high percentage of people living with HIV [8,10]. The proportion of IPI is nearly 50% in industrialized countries, whereas in developing countries, the figure is about 95%, with SSA having the highest prevalence [8,10,11]. Globally, 24% of the entire world suffers from the infections of soil-transmitted helminths [12]. IPI are associated with the impoverished and deprived communities in the populace because of their substandard living conditions, poor access to potable water, extreme poverty, and poor waste disposal systems [13], resulting in anemia, dehydration, vitamin deficiencies, abnormal absorption of nutrients in the gastrointestinal tract, severe persistent diarrhea, malnutrition, retarded growth, and reduced mental development [10,13,14]. The majority of IPI do not exhibit symptoms and, as such, have not been considered as important as bacterial and viral infections in medical importance ranking [14].

The two most prevalent helminths that are transmitted through soil are *Ascaris lumbricoides* and *Trichuris trichiura* [15]. Despite the use of antiretroviral therapy (ART) to reduce the occurrence of diseases that take advantage of weakened immunostatus of HIV patients, rapid episodes of re-infection after deworming are still common because pharmaceutical intervention alone does not prevent reinfection [16]. The continuous ingestion of infective stages of intestinal parasites in an endemic region gives rise to the superposition of existing parasites by a new incoming worm, leading to rapid re-infection following drug treatment [17]. Infection with multiple parasites can cause health defects, especially among HIV patients. The efficiency of some HIV vaccines in HIV patients is affected because treatment of re-infection amplifies drug resistance, affects the efficacy of the front-line drugs, and still permits transmission. To control re-infection, the social factors underpinning the prevention must be controlled [18].

Experimental studies like the WASH (Water, Sanitation, and Hygiene) study promoted hygiene education and showed that disease-free communities require adequate and equitable access to water, sanitation, and hygiene, since treatment alone cannot control the transmission cycle except in situations of very low transmission [19]. In South Africa, treatment of intestinal parasites is usually offered to clinic-attending HIV patients upon suspicion but not routinely for patients being initiated into an antiretroviral program to be screened and dewormed. This has flagged the need to develop an all-inclusive intervention strategy. The Treatment, Hygiene, Education, and Sanitation (THEdS) bundle developed by the team in this study is one of the advocated intervention approaches aimed at promoting healthy behavior among HIV patients on ART in endemic areas. This study, therefore, was conducted as an intervention to mitigate instances of re-infection of intestinal parasites among PLHIV using the THEdS bundle approach.

## 2. Materials and Methods

### 2.1. Study Design and Setting

A community-based non-equivalent control group post-test-only design, an aspect of quasi-experimental design (QED), was conducted from March 2019 to February 2020. The study was carried out in four clinics designated for HIV/AIDS patients. The experimental clinics were the Tsolo Gateway Clinic and Idutywa Health Center, while the control group was drawn from the Ngangalizwe and Nqamakwe Health Centers. These four clinics are located in two Eastern Cape districts, the OR Tambo and Amathole district municipalities (DM). The Ngangalizwe Health Centre and Tsolo Gateway Clinic are in OR Tambo DM while Idutywa and Nqamakwe Health Centres are in Amathole DM. These areas are the traditional home of the indigenous Xhosa people, characterized by underdeveloped infrastructure with people living below the poverty line.

### 2.2. Study Participants

The study participants comprised the cohort that tested positive for intestinal parasites at the first stage of the pre-test assessment. The overall prevalence of intestinal parasitic infection among HIV patients in this population was 30% [20]. These 180 participants formed the cohort who received the treatment and intervention.

### 2.3. Sample Size Calculation

From an unknown population of HIV-positive patients attending various HIV clinics within the study sites covered, sample size (n) was sourced using the following formula:

$$n=\frac{Z_{\propto }^{2}\times P(100-P)}{e^{2}}\;\mathrm{where}P\;(\mathrm{expected}\mathrm{proportion})\mathrm{is}50\%$$

Confidence level (*z*_∝_) is 1.96 and *e* (maximum error admitted) is 4%.

Therefore, by substitution

$$n=\frac{{\left(1.96\right)}^{2}\times 50(100-50)}{4^{2}}=\frac{3.84\times 2500}{16}=600$$

The number of patients that tested positive to intestinal parasites after screening was 180.

These 180 formed the cohort that received treatment.

### 2.4. Data Collection and Sampling Technique

The quantity and quality of data available, as well as the geographic representation of rural and peri-urban areas, led to the purposeful selection of the four clinics designated for the management of HIV/AIDs. Participants who consented were HIV patients who were drawn from the sampling frame of HIV-infected patients attending the identified outpatient clinics between March and December 2019. With the assistance of qualified health care professionals, a comprehensive, anonymous pre-tested questionnaire was utilized to gather clinical data and participant’s fundamental characteristics. Important data were collected after the participants signed informed consent forms. The questionnaire had three sections, namely socio-demographic (place of residence, age, marital status, gender, and education level), socio-economic (family size, number of rooms, types, and number of toilets), and clinical characteristics (CD4 count and viral load). Additionally, two CD4 count and viral load measurements (firstly at baseline and secondly after post-test assessment follow–up) were retrieved from the National Health Laboratory Services (NHLS) Mthatha database. All information was entered into Excel files and checked for correctness, completeness, and consistency. Data confidentiality was ensured using a coding system, and access to the data was restricted.

### 2.5. Sample Collection and Laboratory Investigation Procedures

Stool samples were collected and analyzed to ascertain if there was reinfection (Post-test re-infection). Approximately 5 g of fresh feces (uncontaminated with urine or water) were collected into a clean, dry, wide-mouthed, leak-proof universal bottle. The stool samples were transported in a cold chain system to Walter Sisulu University Medical Microbiology Laboratory, Mthatha campus, for analyses within 5–8 h of collection. Direct stool examination and concentrated methods were used to identify intestinal parasites. Formalin-ether sedimentation and modified Ziehl–Neelsen staining techniques were carried out on the newly collected and submitted stool sample to identify intestinal parasitic infections of protozoa and helminths.

#### 2.5.1. Formol—Ether Concentration Method

The standard procedure for stool sample collection was applied and an amount of preserved stool was processed. In total, 1 g of stool was deposited using an applicator stick into a conical centrifuge tube that contained 7 mL 10% formal water. The suspension was filtered using a sieve before it was transferred into a second conical tube. To the resultant formalin solution three to four milliliters of diethyl ether was added, followed by centrifugation for one min at 2000× *g*. After air-drying, a smear was made and examined under a light microscope with magnifications of 100× and 400×. The supernatant was disposed of thereafter [21].

#### 2.5.2. Modified Ziehl–Neelsen (Z–N) Method

To find opportunistic intestinal parasites, a tiny sample of the concentrated stool material was processed. The sediment of concentrated excrement was used to make a thin smear, which was then left to air dry. The application of methanol to the slides was performed for 1 min to glue the slides, and staining with carbol fuchsin was performed for 30 min. Counterstaining of the slides with 0.3% methylene blue was carried out for 60 s after being decolorized with 1% acid alcohol for 1–2 min and cleaned with tap water. The slides were then examined under a light microscope with a 1000× magnification after being cleaned with tap water and air-dried [21]. The positive slides were cross-checked for oocysts by skilled laboratory personnel for oocysts.

#### 2.5.3. Treatment of Parasitic Infections

Participants infected with intestinal parasitic infections were treated using the standard drugs approved by the World Health Organization (WHO). The clinician was consulted when participants were discovered to have parasitic infections where treatment was given using either of the antiparasitic drugs as follows: Albendazole (400 mg) orally for 3 consecutive days for adults or Mebendazole (500 mg) orally once as a single dose or Mebendazole 300 mg twice daily for 3 days. Combined therapy of Albendazole and Mebendazole was administered quarterly. Metronidazole 500–750 mg orally 3 times daily for 5 days for *B. coli* and Praziquantel 40 mg/kg/a day were given in 2 divided doses for 1 day.

##### THEdS (Treatment, Health Education and Sanitation) Bundle Approach Intervention

The study sites were categorized into two for intervention purposes. The experimental clinics comprised Idutywa Health Centre and Tsolo Gateway Clinic, while the control clinics comprised Ngangalizwe Health Centre and Nqamakwe Health Centre.

Participants from the experimental group received a full intervention package, which included treatment, health education on personal hygiene, and sanitation training, while those from the control group received treatment (only) for intestinal parasitic infections. Both the control and experimental groups received drug administration of albendazole 400 mg, mebendazole 500 mg, single dose/300 mg for 3 days, or combined therapy, which was administered quarterly, as well as metronidazole for *Balantidium coli* and a praziquantel standard dose of 40 mg/kg for *Schistosoma mansoni*. In addition to the treatment, the experimental group were introduced to hygiene education and sanitary practices as interventions. This intervention is called the “THEdS” (Treatment, Health Education and Sanitation) bundle, an acronym coined by the study team. Infrastructure improvement was excluded due to budget.

The intervention involved demonstrating hygienic and sanitary practices such as boiling or chlorinating water before drinking, hand washing with soap before food handling or after visiting the toilets, and general environmental sanitation practices. Posters, fliers, and recorded audio messages on hygiene education and sanitation were also utilized. Sachets of 2.5 g of chlorine tablets and hand washing soaps were distributed. The intervention was aimed at changing the behavior of the HIV/AIDS participants to live a cleaner and healthier lifestyle. The effectiveness of the interventions was assessed through post-test cure rate and re-infection level, odds ratio analysis, and viral load assessment.

##### Post-Test Assessment

This was performed after 21 days of the treatment and intervention. Each participant produced 5 g of freshly made fecal sample for analysis and the outcome was recorded. The effectiveness of the interventions was assessed through post-test cure rate, re-infection, and viral load assessment.

### 2.6. Data Processing and Analysis

SPSS version 27.0 was used to analyze data. Intestinal parasitic prevalence was one of the sociodemographic, clinical, and socioeconomic traits of the respondents that were represented as frequencies and percentages. In order to ascertain the association between the presence of intestinal parasites and the respondents’ sociodemographic, clinical, and/or socioeconomic variables, bivariate analysis was performed by calculating the odds ratio at a 95% confidence interval. A significance criterion of α < 0.05 was established. To determine the efficacy of the THEdS bundle intervention, the odds ratio was computed.

## 3. Results

### 3.1. Socio-Demographic Attributes of Respondents

The 180 HIV patients who were receiving ART were added to the analysis, and 75% were female. The ages of the respondents were from 12 to 75 years, with a mean age of 40.3 (±13.4) years. The majority were single (66.6%), had high school education (66.6%), and dwelled in rural areas (67.7%) (Table 1). Over 50% of the respondents (58.3%) had a family size that is more than four members, with 70.5% having traditional toilets, and those having only one toilet in the house accounting for 82.7%.

Table 1 displays the participants’ demographic information. The sociodemographic characteristics of participants from the experimental and control clinics were similar in cultural and traditional inclinations except for the high proportion of females (more prone to intestinal parasitic infections due to house chores that are related to risk factors), low level of higher education (also a risk factor), type of toilet use, family size, and the locality where the dwelling was located.

### 3.2. Prevalence of Intestinal Parasite

At the baseline level, 14 different intestinal parasites were discovered in 180 HIV/AIDS patients. They were found using a combination of direct wet mounts and smear methods. They are as follows: *Ascaris lumbricoides* (55.9%), *Balantidium coli* (15.1%), *Escherichia coli* (11.3%), *Diphyllobothrium latum* (4.3%), *Taenia* spp. (3.8%), *Schistosoma mansoni* (1.6%), *Cryptosporidium* spp. (1.6%), *Hymenolepsis nana* (1.6%), *Enterobius vermicularies* (1.1%), *Fasciolopsis busiki* (1.1%), *Trichuris trichiuria* (1.1%), *Cystoisopora belli* (0.5%), *Fasciola hepatica* (0.5%), and *Trichostrongylus* species (0.5%) [19].

The experimental clinics located at Idutywa and Tsolo gateway had 29 (16.11%) and 30 (16.67%) participants, respectively, while the control clinics in Ngangelizwe and Nqamakwe had 74 (41.11%) and 47 (26.11%) participants, respectively.

Table 2 shows that out of 180 parasitized participants, only 140 returned for treatment. There was an observed attrition of 22%. The THEdS bundle intervention, which involved drug treatment with hygiene education, was administered to 50 people on experimental sites, while treatment only was administered to control sites with 90 subjects. Control received drug treatment only, while the experimental group received treatment and THEdS bundle intervention.

Table 3 shows that only 62 participants returned after treatment (control) and THEdS intervention (experimental) to submit stool specimens for endline assessment for re-infection. High attrition (55.7%) was recorded here due to the taboo surrounding stool specimen collection.

The presence of multiple parasites occurred at Ngangelizwe clinic and Tsolo clinic. At Ngangalizwe, two male patients had dual infections of both *Ascaris lumbricoides* and *Escherichia coli*, while in Tsolo clinic, one female patient had Ascaris lumbricoides and Balantidium coli. At baseline, IPI with the highest frequency of occurrence was *Ascaris lumbricoides* (n = 29/65), followed by *B. coli* (15/65).

Regarding frequency, the parasitic load was reduced from 65 to 31, significantly reducing *A. lumbricoides* and *B. coli*. *Ascaris* reduced from 29 to 16, while *B. coli* reduced from 15 to 1. The percentages of occurrence are as follows: *A. lumbricoides*, 51.6%; *E. coli*, 32.3%; *Taenia* spp. 9.7%; *B. coli*, 3.2%; and *Crytosporidium parvum*, 3.2%. For an unknown reason, *E. coli* increased while *Taenia* spp. remained unchanged. A new parasite, *C. parvum,* was detected.

Table 4 compares the pre-test (pre-intervention) and the post-test (post-intervention) stool assessment.

The table shows that out of 12 parasites, 7 types of parasites were cleared after THEdS. However, *C. parvum*, which was initially not among the initial parasites, was detected at the reinfection stage.

Table 5 shows the cure and re-infection proportions in the control and experimental sites after the final assessment. The reinfection was highest at Nqamakwe clinic (50%), a rural control site, while reinfection was lowest at Tsolo clinic (35.7%), a rural but an intervention site. The overall cure (experimental and control) was 54.8%. The overall reinfection proportion was 45.2%. The total number of patients cured was 34/62. The total number of patients reinfected was 28/62.

Experimental sites: the cure proportion was 60%, while re-infection was 40%.

Control sites: the cure rate was 51.4%, and recurrent infection was 48.6%.

The odds ratio of parasitic cure proportions among intervention clinics compared with control clinics is shown below:
Odds ratio = (odds of parasitic cure proportion among intervention clinics)/(odds of parasitic cure proportions among control clinics).
Calculation of odds ratio for clearing the parasite after intervention compared to the control group.
Odds of parasite clearing amongst intervention group/odds of parasite clearing amongst control group = (15/10)/(19/18) = 1.42 (95% confidence interval (CI): 0.51–3.92),

indicating that the intervention group is 1.42 times more likely to have parasite clearing than the control group. However, the CI overlaps 1, indicating that it is not statistically significant, although it would be inappropriate to interpret it based only on the CI. The *p*-value = 0.502 (χ^2^ = 0.45, df = 1) is greater than the level of significance (α = 0.05), so we can say there is no evidence against the null hypothesis, indicating that there is no discernible variation in the likelihood of parasite clearing within the control group and the intervention group.

The relative risk or risk ratio (RR) calculation is as follows:
risk of parasite clearing amongst intervention group/risk of parasite clearing amongst control group = 1.17.


The relative risk or risk ratio (RR) when the outcome is cleared of parasites = 1.17 (CI: 0.75–1.82), indicating that one is 1.17 times more likely to get cleared of the parasite if there is no intervention. The CI includes 1, which suggests the RRs are not significant. The *p*-value = 0.502 (χ^2^ = 0.45, df = 1) is greater than the level of significance (α = 0.05), so we can say there is no evidence against the null hypothesis suggesting that there is no significant difference in the risk of a parasite not clearing within the intervention group and the control group.

Table 6 shows the mean and CI (95%) for the viral load of the HIV patients before and after intervention. The viral load decreased from 377 to 44.

## 4. Discussion

In developing countries, intestinal parasitic infections continue to be a serious threat to public health in less industrialized nations, particularly in immune-compromised patients and vulnerable individuals like HIV-infected patients in SSA, which has the highest HIV prevalence [11,22]. HIV-infected patients have experienced reprieve since the introduction of the widely obtainable ART [11], nevertheless, opportunistic infections including IPI decreased their quality of life resulting in illnesses and death [23,24]. The development of targeted interventions in this vulnerable population is imperative to enhance the general health status of HIV patients.

The majority of the participants were females (75%), which might imply the low attendance of men at the HIV clinics. It has been noted that in Africa, a higher percentage of men do not seek medical treatment. The culture of “tough” masculinity, which portrays males as the stronger gender and less vulnerable than women, has been blamed for this [3]. Due to the opportunities for HIV testing via reproductive health services for women, they are thus able to present early enough for treatment even while asymptomatic [25]; While men seek treatment late particularly when symptoms are at the advanced stage [26].

The participants’ sociodemographic characteristics show high propensity risk factors for helminthic infections. As many as 149/180 (82.7%) respondents had one traditional pit latrine cubicle in their households. This predisposes the family to IPIs. Despite knowledge of helminthic transmission, the use of limited toilet facilities for a large number of respondents predisposes such families to infection with intestinal parasites transmitted in a fecal-oral cycle. The study in Chad found that 92.8% of participants had a traditional toilet facility, while 2.9% have modern toilet facilities [27]. Comparable to our findings is the result of a community-based survey by Mfenyana et al. [28] that showed 17.9% of family units used flush toilets.

Of all 180 participants, baseline microscopic examination of stool samples identified 14 species comprising helminths and protozoa. The most commonly found parasites were *A. lumbricoides* (55.9%) and *B. coli* (15.1%). This agrees with the studies conducted by Gimba and Dawam [29], and Gizaw et al. [30] in Nigeria and Ethiopia, respectively, who found *A. lumbricoides* as the most common enteric parasite. Another study conducted in Mozambique reported that the most frequently detected helminths in both HIV-infected and uninfected populations were *T. trichiura* (12.9%), and *A. lumbricoides* (5.2%) [10]. However, in the studies of Karim et al. [31] and Aschale et al. [12] conducted in Pakistan and Ethiopia, respectively, *E. histolytica* was the most detected among intestinal parasites, while Gholipoor et al. [32] in Iran found the most prevalent to be *G. lamblia* (*Giardia duodenalis*). The fecal–oral pathway remains the route through which these parasites are spread. According to Karim et al. [31], the risk of ingesting the parasite is high because farm products are cultivated using sewage water. The presence of mixed parasitic infection and bacteria (*E. coli*) as seen in our study was corroborated by other studies. Unlike ours, other studies have reported mixed parasitic infections of up to five parasites [33]. The study of Cerveja et al. [10] reported mixed infection with different combinations of *T. trichiura* and *A. lumbricoides* (8.1%) and *T. trichiura* with *E. coli* (4.7%), while our study only had mixed infection comprising parasites and bacteria, A. *lumbricoides* with *E. coli (bacteria)*, and *A. lumbricoides* with *B. coli*. These differences could mean that our HIV-infected participants were less immunologically advanced and had improved living conditions than others. According to Cerveja et al. [10], HIV-infected patients had fewer mixed infections, (15.3%) than HIV-uninfected patients (28%), however, the difference did not reach statistical significance. In our study, we did not include HIV-uninfected patients for comparison. The results of this study indicated that IPI is still a substantial public health problem in the Eastern Cape and should be monitored.

Though the complex relationship between human and their parasites may not be clearly understood, the dynamic pattern of infection and reinfection in humans with predictable phenomena varies with geographic region, socio-economic status, personal hygiene, and behavioral changes. Preventive programs that use a combination of different strategies offer the most efficient approach for people with exposure and susceptibility to intestinal parasites. A broad intervention control strategy known as THEdS was designed and implemented to reduce infection and prevent reinfection of parasitic disease in person in PLWHA in selected parts of Eastern Cape, South Africa.

Only 62 participants returned AFTER treatment only (control) and THEdS intervention to submit stool specimens for endline assessment to ascertain re-infection. High attrition (65.6%) was recorded here due to the taboo surrounding stool specimen collection.

Of the 12 intestinal parasites (IPs) isolated from the stool samples, *Ascaris lumbricoides* had the highest frequency of reinfection. The high prevalence of *A. lumbricoides* agrees with the study conducted by Pawestri et al. [34] in an underdeveloped neighborhood in the Thai–Myanmar area with a similar setting, where *A. lumbricoides* ranked high (45.8%) among the intestinal parasites isolated. Though the actual data on prevalence, risk factors, and specific interventions to reduce helminthic infections and their reoccurrence are scarce and probably under-reported in both settings, the findings of this study and others [35] demonstrate the endemicity of *A. lumbricoides* among deprived communities with lack of basic education on the transmission of intestinal parasites, inadequate potable water supply, and poor personal hygiene.

Our findings on the comparison between the pre-test (pre-intervention) and the post-test (post-intervention) stool assessment demonstrated that 7 out of 12 parasites isolated were cleared. However, *Cryptosporidium parvum* which was initially not isolated was isolated at the reinfection stage.

The study’s approach utilized the THEdS bundle, as an intervention to inculcate in the participants a personal responsibility to cultivate hygienic practices that can sustain the therapy being administered while preventing reinfection. This strategy is long-term sustainable in controlling IPIs in this vulnerable population. The effectiveness of the THEdS bundle intervention is demonstrated by the higher cure proportion in experimental clinics in contrast to the control clinics. Most IPs were detected in control clinics at the post-test, with “treatment only” administered. This is an indication that the intervention bundle was successful despite non-statistical significance.

The significant decline in the viral load after the THEdS intervention is corroborated by the study of Walson et al. [36], who suggested that treating parasites, particularly helminths, in HIV/AIDS patients may reduce the plasma viral load. Their study involved albendazole therapy, which significantly increased the CD4 counts and reduced HIV-1 RNA levels among individuals with *A. lumbricoides* infection after 12 weeks of follow-up. Our study used an integrated intervention approach combining therapy with hygienic practices, which makes it more impressive. The reduction in viral load and increase in CD4 count, which are statistically significant, are both indications of a very successful intervention.

One of the limitations of this study was obtaining stool samples for the second phase of testing for reinfection. In this part of the world, it is believed that a person’s stool is private to him/her and if handed out to others, can be used to manipulate an individual negatively. Despite consent, high attrition was noticeable during post-test assessment.

## 5. Conclusions

This study demonstrates the effectiveness of a comprehensive, integrated intervention approach that successfully reduced intestinal parasite infections and its reinfection in compromised individuals. The findings show that the intervention increased the participant’s knowledge of how intestinal parasites are transmitted, and how to sustain hygienic living to reduce the rate of infection and reinfection with these parasites.

The odds ratio indicated that the intervention group is 1.42 times more likely to be clear of parasites as compared to the control group. Further findings show that this integrated intervention of treatment and hygienic practices among HIV-infected patients reduced the plasma viral load and increased the CD4 counts. The THEdS strategy could be a model for sustainable control and prevention of intestinal parasites and other diseases whose transmission is associated with environmental and food contamination and poor personal hygiene.

## Figures and Tables

**Table 1:** Demographic characteristics of participants.

Variable	n	(%)
Gender	180	
Male	45	(25.0)
Female	135	(75.0)
Age (years)	179	
12–20	14	(7.8)
21–30	30	(16.7)
31–40	58	(32.2)
41–50	42	(23.3)
51–60	20	(11.1)
61–70	14	(7.8)
>70	1	(0.6)
Education level		
Primary	47	(26.1)
Secondary	120	(66.6)
Tertiary	12	(6.6)
Other	1	(0.5)
Marital status		
Single	120	(66.6)
Married	51	(28.3)
Divorced	3	(1.6)
Widowed	6	(3.3)
Residence		
Rural	122	(67.7)
Peri-urban	58	(32.2)
Family size		
1	11	(6.1)
2	13	(7.2)
3	30	(16.6)
4	21	(11.6)
>4	105	(58.3)
Number of rooms		
1	46	(25.6)
2	26	(14.4)
3	30	(16.7)
4	34	(18.9)
>4	44	(24.4)
Type of toilet		
Traditional	127	(70.5)
Modern	48	(26.6)
Both	3	(1.6)
Bush	2	(1.1)
No of toilets		
1	149	(82.7)
2	28	(15.5)
3	1	(0.5)
None	2	(1.1)

**Table 2:** Treatment for intestinal parasites in the control and experimental groups (n = 140).

Sites	Number Infected	Treatment Only and Treatment + THEdS
Experimental	29	25 (17.86%)
Control	74	53 (37.86%)
Control	47	37 (26.42%)
Experimental	30	25 (17.86%)
Total	180	140 (100%)

**Table 3:** Endline post-intervention assessment of stool specimen to establish reinfection (n = 62).

Sites	Infected Participants	Endline Stool Assessment
Experimental	25	11 (17.74%)
Control	53	25 (40.32%)
Control	37	12 (19.36%)
Experimental	25	14 (22.58%)
Total	140	62 (100%)

**Table 4:** Comparison of the pre-test (pre-intervention) and the post-test (post-intervention) stool assessment.

	Baseline Parasitic Load (Before THEdS)		Endline Parasitic Load (After THEdS)
Parasites	Sample Size N	Frequency n (%)	Sample Size After Intervention n (%)
*Ascaris lumbricoides*	29	29 (44.6)	16 (51.6)
*Balantidium coli*	14	15 (23.1)	1 (3.2)
*Escherichia coli*	6	8 (12.3)	10 (32.3)
*Taenia* spp.	3	2 (3.1)	3 (9.7)
*Diphyllobothrium latum*	2	3 (4.6)	0
*Fasciolopsis busiki*	2	2 (3.1)	0.0
*Enterobus vermicularies*	1	1 (1.5)	0.0
*Schistosoma mansoni*	1	1 (1.5)	0.0
*Trichostrongylus* spp.	1	1 (1.5)	0.0
*Trichuris trichiura*	1	1 (1.5)	0.0
*Hymenolopsis nana*	1	1 (1.5)	0.0
*Cystoisospora belli*	1	1 (1.5)	0.0
*Crytosporidium parvum*	0	0	1 (3.2)
	62	65 (100)	31 (100)

**Table 5:** Cure proportions versus reinfection rates in experimental and control group.

Experimental Clinics				Control Clinics			
Clinic	Infected	Cured n (%)	Reinfection n (%)	Clinic	Infected	Cured n (%)	Reinfection n (%)
Idutywa clinic	11	6 (40%)	5 (50%)	Ngangalizwe clinic	25	13 (52)	12 (48)
Tsolo clinic	14	9 (60%)	5 (50%)	Nqamakwe clinic	12	6 (50)	6 (50)
Total	25	15 (60%)	10 (40%)	Total	37	19 (51.4)	18 (48.6)

**Table 6:** Assessing the effectiveness of the THEdS intervention using patients’ viral load (n = 62).

Viral Load	Mean (S.D)	CI (95%)	* *p*-Value
Baseline	377 (376.3)	242.3–513.7	0.000
After intervention	44 (60.5)	22.2–65.8	

* *p*-value for paired samples *T*-test; S.D = standard deviation; CI = confidence interval.

## Data Availability

Data can be requested from the corresponding author.
